# Mortality and reoperations following treatment of acetabular fractures in patients ≥ 70 years: a retrospective cohort study of 247 patients

**DOI:** 10.2340/17453674.2024.42704

**Published:** 2025-01-20

**Authors:** Johan LJUNGDAHL, Björn HERNEFALK, Anna PALLIN, Anders BRÜGGEMANN, Nils P HAILER, Olof WOLF

**Affiliations:** Department of Surgical Sciences, Section for Orthopaedics, Uppsala University, Uppsala, Sweden

## Abstract

**Background and purpose:**

Evidence for long-term outcomes following acetabular fractures in older adults is limited. We aimed to evaluate mortality, complications, and need for subsequent surgical procedures in operatively and nonoperatively treated older patients with acetabular fractures.

**Methods:**

Patients aged ≥ 70 years with acetabular fractures treated at Uppsala University Hospital between 2010 and 2020 were included. Fractures were classified according to Letournel. Local medical records were analyzed and cross-referenced with the Swedish Arthroplasty Register to identify reoperations and delayed arthroplasty procedures. Follow-up time ranged from 2–12 years. Primary outcome was mortality 1 year after injury. Descriptive statistics, survival analysis using the Kaplan–Meier method, and logistic regression models were used.

**Results:**

247 patients (67% men) with a median age of 80 years (range 70–102) were included. Most patients were ASA class 3 (67%). 148 (60%) patients were treated operatively. The 1-year mortality was 15% (95% confidence interval [CI] 9–21) in the operatively and 29% (CI 19–37) in the nonoperatively treated group. Difference in adjusted mortality rates between treatments did not reach statistical significance. 20% of patients treated with open reduction internal fixation (ORIF) underwent some form of reoperation. In the nonoperatively treated group, 1% had a delayed THA.

**Conclusion:**

The 1-year mortality following acetabular fractures in older people was 21% (CI 15–26), underscoring the frailty of this patient group. ORIF alone was associated with a 20% reoperation rate while the rate of delayed surgical treatment in patients selected for nonoperative treatment was 1%.

The incidence and mean age of individuals diagnosed with acetabular fractures have shown a notable rise over the past 20 years, with men being more susceptible compared with women [1-3]. Acetabular fractures in the geriatric population are often fragility fractures sustained by same-level low-energy falls [4-7].

Treatment options in geriatric acetabular fractures range from nonoperative management to operative treatment with either open reduction and internal fixation (ORIF), total hip arthroplasty (THA) alone or in combination with ORIF – also referred to as a combined hip procedure (CHP), or “fix and replace” [8].

Little is known about mortality in acetabular fractures in older people, especially following nonoperative treatment [7]. Also, studies including both ORIF, primary THA, CHP, and nonoperative treatment are scarce [9]. The overall 1-year mortality is reported as up to 24% in patients > 70 years [10], which is comparable with patients with femoral fractures [11]. There is a lack of studies with larger study populations and comparisons between treatment groups are also often difficult due to selection bias.

We primarily aimed to compare mortality rates in patients aged ≥ 70 years with acetabular fractures treated operatively or nonoperatively, specifically the potential impact of primary THA/CHP on mortality compared with ORIF and nonoperative treatment. Secondarily, we aimed to describe complications and the burden of secondary surgical treatment between treatment regimes.

## Methods

### Study design, setting, and participants

This retrospective cohort study was based on medical record data from Uppsala University Hospital. The data was linked to the Swedish Arthroplasty Register (SAR) for information on secondary arthroplasty procedures. As of 2021, the register boasts a coverage rate of 98.1% for primary THA and 93.5% for revision THA [12]. The patient’s unique identification number (PIN), given to all people registered in Sweden, was used to review medical records, radiographs, and subsequent linkage to SAR. The study is reported in accordance with the STROBE guidelines [13].

All patients ≥ 70 years at the time of injury with an acetabular fracture (S32.4 in the International Classification of Diseases-10 [ICD-10] [14]) treated at Uppsala University Hospital between January 2010 and February 2020 were identified in the hospital’s medical records and included in the study. Patients were either residents of Uppsala County or referrals for operative treatment at Uppsala University Hospital, the regional referral center for these injuries.

### Radiographic assessment

2 of the authors (JL and BH) independently classified the preoperative radiographs using the Letournel classification. Classifications were then compared and, if inconsistent, mutual agreement was set after a joint review. Raters primarily agreed on 79% of the fracture patterns reviewed and discussed the remainder for final classification. Computer tomography was available in most cases and, when missing, plain radiographs or magnetic resonance images (MRIs) were used. Displacement ≥ 2 mm and acetabular cranial or medial roof impaction (Gull sign) were assessed as present. After radiographic assessment, 49 patients were deemed ineligible for the study. These exclusions included 16 cases of implant-related fractures, 15 cases of fractures to the pubic rami, 11 cases of pelvic ring injuries, and 4 cases of pathological fractures. 2 patients had missing radiographs, and 1 had an incomplete fracture diagnosed only after undergoing an MRI assessment. Upon a review of medical records, it was ascertained that 1 patient was treated at another hospital, and was thereby excluded from the study.

### Study variables

Medical records were reviewed to assess patient factors, the mechanism of injury, and the type of treatment. Baseline variables were age, sex, cognitive function, comorbidity, residence, and ambulatory status before the injury. Comorbidity was classified according to the American Society of Anesthesiologists (ASA) physical status classification system [15], either as documented by the anesthesiologist in the medical record, registered in the SAR, or assessed according to the Swedish translation of the ASA physical status classification system. The patient’s residence at the time of injury was categorized into 4 categories: private residence, private residence with elderly care service, elderly care accommodation, or unknown. Pre-injury ambulatory status was divided into 5 categories: no walking aid, crutches, use of a walker, prosthesis after amputation, non-walker and unknown. The mechanism of injury was categorized into 6 groups: same-level falls, falls from other levels, unspecified falls, bicycle accidents, other transportation accidents, or unknown.

Treatment was categorized into operative (ORIF/THA/CHP) or nonoperative. Operative treatment was defined as surgical treatment within 30 days after injury. Secondary treatment was set as operative treatment > 30 days after injury and included both operative treatment after failed nonoperative treatment and reoperations after primary operative treatment. Secondary treatment also included reoperations within 30 days from injury.

### Clinical routine

Type of treatment depended on fracture pattern, presence of displacement, and impaction (i.e., Gull sign), as well as patient comorbidity and mobilization status. The consultants present when radiographs and medical history were presented mutually agreed on a treatment plan. During the study period there was a gradual shift towards CHP from ORIF. ORIF was typically performed using the modified Stoppa approach and a suprapectineal plate to stabilize anterior structures. The Kocher–Langenbeck (K–L) approach was used to gain access to posterior column fractures. When a THA was indicated in the acute setting, we routinely employed a direct lateral approach to insert a Burch–Schneider type reinforcement ring after using the patient’s femoral head for bone graft impaction. A dual mobility cup was then cemented into the Burch–Schneider ring. A cemented anatomical stem was standard for the femur. Our institution defines the CHP procedure as combining ORIF and THA in the same surgical session. Depending on pathology, the Stoppa and direct lateral approaches were combined after repositioning, or the K–L approach was used for the entire procedure. All patients received analgesia and thromboembolic prophylaxis. After THA and CHP treatment, patients were permitted to engage in immediate full weightbearing, whereas patients with ORIF were mobilized with partial weightbearing if possible. Patients who were treated nonoperatively were routinely given analgesia and mobilized initially without weightbearing allowed on the injured side. Repeat radiographs were taken after 1–2 weeks to evaluate fracture position, and full weightbearing was generally allowed after 6–8 weeks.

### Study outcomes: mortality, complications, and secondary treatment

The primary outcome was mortality, assessed after 30, 90, and 365 days. Any date of death was found either in the local medical records or in SAR. Both sources are linked to the Swedish Tax Agency, the authority responsible for registering deaths in the population register.

Secondary outcomes were complications and secondary treatment. Local medical records were reviewed for complications, divided into neurological injury, vascular injury, infections, hip joint failure, and THA dislocation. Infections were subdivided into surgical site infection (SSI) and periprosthetic joint infection (PJI). Hip joint failure was further categorized into avascular necrosis of the femoral head (AVN), acetabular protrusion, and posttraumatic osteoarthritis.

Medical records were reviewed for secondary treatment, defined as subsequent surgical procedures, i.e., operative treatment after failed nonoperative treatment or reoperations after failure of primary operative treatment. Details were noted for late primary THA (after failed nonoperative treatment), conversion to THA (after ORIF), THA revision, wound debridement, and debridement, antibiotics, and implant retention (DAIR). The cause and date for the procedure were noted. The cohort was then linked to the SAR to identify procedures performed elsewhere with a follow-up time between 29 months and 12 years (SAR data extracted July 22, 2022). We were not able to review medical records from other hospitals. Therefore, as for complications not leading to further surgery, follow-up time was limited to date of discharge from our hospital regarding referred patients, although for more serious complications the referral hospitals usually contact us for advice.

### Statistics

Descriptive statistics were used to present the baseline data. Continuous data was presented as medians with ranges and categorical data as proportions. Survival analysis was performed using the Kaplan–Meier method. Cox regression models adjusted for sex, age, and ASA class were fitted to estimate the risk of death with 95% confidence intervals (CIs). Statistical analyses were performed using R Studio version 4.3.1 (R Foundation for Statistical Computing, Vienna, Austria).

### Ethics, registration, funding, and disclosures

The Swedish Ethical Review Authority approved this study on September 15, 2021 (Dnr: 2021-04153). No funding was received. No conflicting interests were declared. Complete disclosure forms for this article following the ICMJE template are available on the article page, doi: 10.2340/17453674.2024.42704.

## Results

**Figure 1:**
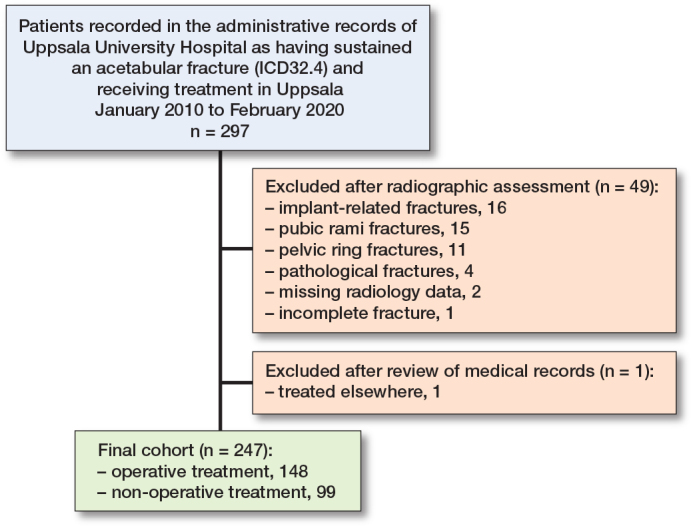
Patient flowchart.

297 patients ≥ 70 years with an acetabular fracture were found in our hospital’s medical records (Figure 1). 50 patients were excluded after radiographic assessment or review of medical records. 247 patients were finally included in the study: 148 treated operatively and 99 treated nonoperatively.

### Baseline characteristics

The 247 included patients had a median age of 80 years (range 70–102) (Table 1). 67% were men. Injury mechanisms were similar between the operative and nonoperative treatment groups, with same-level falls accounting for the injury in over 60% of all cases. The patients who underwent operative treatment were mainly classified as ASA class 3 (59%) and resided in their homes without elderly care services. The majority of them did not require any walking assistance before the injury. In the nonoperatively treated group, 79% were classified as ASA class 3, and cognitive dysfunction was present in 26%, compared with 4.7% in the operatively treated group.

**Table 1:** Demographics of the study cohort of 247 patients aged ≥ 70 years with acetabular fractures and fracture pattern distribution. Values are n (% within group) unless otherwise specified

Characteristic	Operative	Nonoperative	Overall
Patients	148	99	247
Age, median	78	84	80
range	70–95	70–102	70–102
Sex			
Female	46 (31)	35	81 (33)
Male	102 (69)	64	166 (67)
Cognitive dysfunction	7 (4.7)	26	33 (13)
ASA class			
1	10 (6.8)	1	11 (4.5)
2	46 (31)	18	64 (26)
3	87 (59)	78	165 (67)
4	5 (3.4)	2	7 (2.8)
Residence			
Private, no ECS	111 (75)	45	156 (63)
Private, with ECS	20 (14)	24	44 (18)
Elderly care accommodation	9 (6.1)	30	39 (16)
Unknown	8 (5.4)	0	8 (3.2)
Mobilization status			
No aid	84 (57)	32	116 (47)
Crutches	7 (4.7)	3	10 (4.0)
Walker	33 (22)	59	92 (37)
Non-walker	0 (0.0)	5	5 (2.0)
Prosthesis	1 (0.7)	0	1 (0.4)
Unknown	23 (16)	0	23 (9.3)
Injury mechanism			
Bicycle accident	8 (5.4)	3	11 (4.5)
Fall, other level	26 (17)	11	37 (15)
Fall, same level	85 (58)	69	154 (62)
Fall, NOS	11 (7.4)	14	25 (10)
Transportation accident	17 (11)	0	17 (6.9)
Unknown	1 (0.7)	2	3 (1.2)
Fracture patterns			
Elementary fractures	32 (22)	59	91 (37)
Posterior wall	8 (5.4)	4	12 (4.9)
Posterior column	1 (0.7)	2	3 (1.2)
Anterior wall	10 (6.8)	22	32 (13)
Anterior column	11 (7.4)	30	41 (17)
Transverse	2 (1.4)	1	3 (1.2)
Associated fractures	116 (78)	40	156 (63)
Transverse + posterior wall	6 (4.1)	4	10 (4.0)
T-type	11 (7.4)	5	16 (6.5)
Posterior column + post. wall	5 (3.4)	0	5 (2.0)
ACPHT	46 (31)	14	60 (24)
Both column	48 (32)	17	65 (26)
Radiographic assessment			
Displaced fractures	146 (99)	56	202 (82)
Impacted fractures	129 (87)	34	163 (66)
Treatment			
Operative	148(100)		–
ORIF	94 (64)		94 (38)
THA	9 (6.1)		9 (3.6)
CHP	45 (30)		45 (18)
Nonoperative	–	99	99 (40)

ASA class: American Society of Anesthesiologists Physical Status system. ECS: elderly care service. NOS: not otherwise specified. ACPHT: anterior column posterior hemitransverse. ORIF: open reduction internal fixation. THA: total hip arthroplasty. CHP: combined hip procedure (ORIF + THA).

### Fracture classification

Overall, associated fracture patterns were most common (63%), where anterior column and posterior hemitransverse (ACPHT) or both-column patterns accounted for half of all cases (Table 1). Elementary fractures to the posterior wall or column were rare (4.9% and 1.2%, respectively). In the operatively treated group, associated fracture patterns accounted for 78% with ACPHT and both-column injuries making up for almost two-thirds of the cases. In contrast, in the nonoperative group, elementary fracture patterns accounted for 60%, with fractures to the anterior wall or column being present in over 50% of cases.

### Treatment

148 (60%) underwent operative and 99 nonoperative treatment. The operatively treated group had a median age of 78 years (range 70–95) compared with 84 years (70–102) for the nonoperatively treated group. Of the patients selected for operative treatment, 94 underwent ORIF, 9 had a THA alone, and 45 underwent CHP. The ORIF group had a median age of 76 years (70–95) compared with 84 (71–92) for THA and 81 (70–91) for CHP (Table 2). ORIF was the most common operative method for fractures classified as ACPHT or both-column injuries (Table 3). 67% of all CHP cases were classified as T-type, ACPHT or both-column injuries.

**Table 2:** Characteristics of 148 operatively treated patients with acetabular fractures for the different operative methods. Values are count (%) unless otherwise specified

Characteristic	ORIF	THA	CHP	Overall
Patients	94	9	45	148
Age, median	76	84	81	78
range	70–95	71–92	70–91	70–95
Men	73	4	25	102 (69)
ASA class				
1	9	0	1	10 (7)
2	30	3	13	46 (31)
3	52	5	30	87 (59)
4	3	1	1	5 (3)
Cognitive dysfunction	4	0	3	7 (5)
Impaction	81	9	39	129 (87)
Displaced	93	9	44	146 (99)

For abbreviations, see Table 1.

**Table 3:** Distribution of fracture patterns across the 3 types of operative treatments. Values are count

Type	ORIF (n = 94)	THA (n = 9)	CHP (n = 45)
Elementary fractures			
Posterior wall	4	0	4
Posterior column	0	0	1
Anterior wall	5	3	2
Anterior column	7	2	3
Associated fractures			
Transverse + posterior wall	4	0	1
T-type	3	1	7
Posterior column + posterior wall	1	0	4
ACPHT	36	2	8
Both columns	32	1	15

For abbreviations, see Table 1.

### Mortality

**Figure 2:**
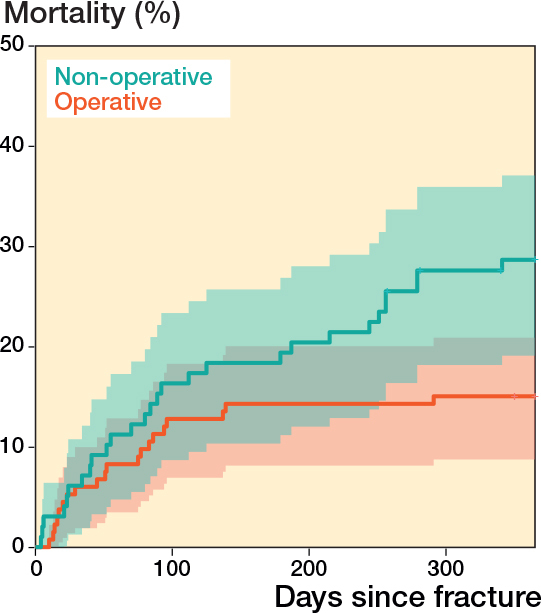
Kaplan–Meier plot illustrating unadjusted mortality in 247 patients aged ≥ 70 years during the first year following an acetabular fracture: operative versus nonoperative treatment including 95% confidence intervals in shaded colors

The 1-year mortality for the entire cohort was 21% (CI 15–26) (Table 4); 15% (CI 9–21) in the operatively treated group and 29% (CI 19–37) in the nonoperatively treated group (Figure 2). The hazard ratio for mortality at 1 year, adjusted for age, sex, and ASA class, was 0.92 (CI 0.49–1.7) for the operatively treated group, with the nonoperatively treated group as the reference. There was also no significant difference in mortality between operatively and nonoperatively treated groups at 30 or 90 days (Table 4).

**Table 4:** 30-, 90-, and 365-day unadjusted mortality calculated using Kaplan–Meier method for operative and nonoperative treatment groups. Values are percentage mortality with 95% confidence intervals

Follow-up	Operative (n = 148)	Nonoperative (n = 99)	Total (n = 247)
30-day	6 (2–10)	6 (1–11)	6 (3–9)
90-day	11 (6–16)	15 (8–22)	13 (8–17)
365-day	15 (9–21)	29 (19–37)	21 (15–26)

### Complications and reoperations

**Figure 3:**
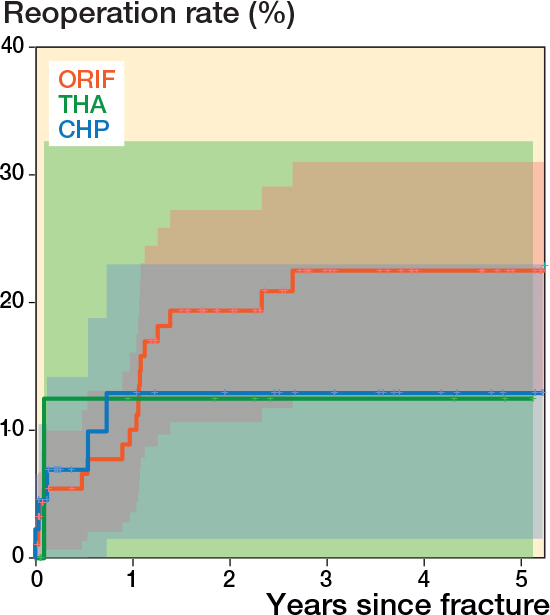
Reoperations within 5 years following acetabular fractures. Kaplan–Meier survival curve including 95% confidence intervals in shaded colors. ORIF: open reduction and internal fixation. THA: total hip arthroplasty. CHP: combined hip procedure (ORIF + THA).

Complications were observed in 41 patients (17%), 38 (26%) in the operatively treated group and 3 (3%) in the nonoperatively treated group (Table 5). Secondary surgical treatment was undertaken in 25 (17%) in the operative group and 1 (1%) in the nonoperative group (Table 6). Reoperation was primarily prompted by the occurrence of posttraumatic osteoarthritis. All reoperations occurred within 3 years of primary surgery (Figure 3).

**Table 5:** Number of complications in 247 patients treated for acetabular fractures in operative and nonoperative treatment groups

Complication	Treatment		Overall (n = 247)
	Operative (n = 148)	Nonoperative (n = 99)	
Neurological	5	0	5
Vascular	3	0	3
Infection	10	0	10
Wound infection	6	0	6
Prosthetic joint infection	4	0	4
Hip joint failure	19	3	22
AVN	3	1	4
Protrusion	4	2	6
Posttraumatic osteoarthritis **^a^**	12	0	12
Total hip arthroplasty dislocation	2	0	2
Total	39 **^b^**	3	42

AVN: avascular necrosis of the femoral head.

a Radiographically diagnosed.

b 1 patient had 2 complications.

**Table 6:** Secondary treatment for each treatment type

Treatment	n	THA conversion	THA revision	DAIR	Wound debridement	Packing	Delayed THA
Operative	148	14	2	4	4	1	
ORIF	94	14			4	1	
THA	9			1			
CHP	45		2	3			
Nonoperative	99						1

For abbreviations, see Table 1 and DAIR: debridement, antibiotics, implant retention.

#### Complications and reoperations in the operatively treated group

1 patient had more than 1 complication; a perioperative bleeding requiring packing and a later SSI. 6 (4%) patients had SSIs and 4 (3%) PJIs. 19 patients (13%) had postoperative hip joint failures. Of the 25 patients requiring reoperation, 5 had more than 1 reoperation. Looking into the different operative methods, 19 patients treated with ORIF had a reoperation, divided into 14 THA conversions, 4 wound debridements, and 1 packing due to bleeding. Of the 14 THA conversions, 11 were due to posttraumatic osteoarthritis and 3 due to AVN. The 4 ORIF patients who had wound debridements were all because of SSIs. 1 of the patients with initial THA had a PJI and underwent a DAIR procedure 5 weeks after initial surgery. Of 45 patients treated with CHP, 5 had reoperations. 2 patients underwent revision surgeries due to dislocations, while 3 patients underwent DAIR procedures for the treatment of PJI. There was a trend toward a higher reoperation rate in the cohort treated with ORIF compared with CHP, but this observation did not reach statistical significance.

#### Complications and reoperations in the nonoperatively treated group

In the nonoperatively treated group, 1 patient developed acetabular protrusion and 2 patients AVN. No posttraumatic osteoarthritis was diagnosed in this group. The only patient who was initially treated nonoperatively and who required secondary surgical treatment had a THA performed 9 weeks post-injury due to acetabular protrusion.

## Discussion

In this study we aimed to describe mortality following operative and nonoperative treatment in elderly people with an acetabular fracture. Furthermore, we examined rates of complications and reoperations. Our primary observations revealed a high mortality rate regardless of treatment type. We believe we found a clear clinical mortality difference, although the adjusted 1-year mortality did not show a statistically significant difference between treatment groups. Mortality rates were comparable to that earlier observed for femoral fractures [11]. Secondary treatment, most commonly conversion to THA, was likely to occur within 2 years, and patients initially treated nonoperatively were rarely subject to delayed surgery.

The finding of a male predominance in our patients is supported by previous studies on older patients with acetabular fractures [2,3,5] but stands in contrast to the sex distribution in hip and pubic rami fractures [3,16,17]. The cause for male predominance is not clear and likely multifactorial. One explanation could be that men have a lower incidence of osteoporosis and a higher bone mineral density in the proximal femur than women [18]. As a result, a fall on the hip may not lead to a hip fracture but transfers the force through the hip joint to the acetabulum. There are also anatomical differences between sexes that could be part of the explanation. Men have larger femoral heads and somewhat more shallow acetabulae than women, decreasing the capabilities of load-sharing and thereby possibly making men more susceptible to fractures of the acetabulum [19].

In our study, nearly two-thirds of all injuries were attributed to same-level falls, a finding that is consistent with prior research [1,2,6]. This further emphasizes the senior populations as a distinct at-risk group for acetabular fractures.

The predominant fracture patterns observed in our study were associated, representing 78% of fractures in the operative group and 63% in the nonoperative group. Ferguson et al. radiographically evaluated 235 patients ≥ 60 years with displaced acetabular fractures receiving operative treatment [2]. In alignment with our results, they predominantly identified associated fracture patterns (63%), with the ACPHT and both-column patterns most frequently observed. In a Swedish study that included 2,132 acetabular fractures from the Swedish fracture register, the same fracture patterns were predominantly treated operatively [10].

In the operatively treated cohort of acetabular fractures, ORIF was the most frequently selected treatment option, accounting for 64% of cases over the study period. Patients treated with ORIF were somewhat younger than those treated with THA or CHP. Younger age is associated with better bone quality and reduced osteoarthritis severity, rendering ORIF a more appealing option in these cases, with the primary ambition being the preservation of the native hip joint. However, our results highlight the risk of posttraumatic osteoarthritis, which may be subject to future THA conversion.

We found only 9 patients who underwent THA as the primary treatment. The standard procedure in our department for these cases is to employ the CHP. Despite the potential benefits of acute THA for acetabular fractures in older patients with compromised bone stock [20], a diminished functional outcome is seen in this demographic group compared with younger patients [21].

The CHP has gained increased attention over the past decade and previous studies have shown lower short-term reoperation rates than ORIF alone, with no associated increase in mortality [8,22]. In our cohort, the CHP was mainly performed in associated fracture patterns, posing challenges to primary THA treatment alone, as acetabular reconstruction becomes difficult without supplemental ORIF.

We found a clinical difference in the 1-year mortality between operative and nonoperative treatment (15% and 29%, respectively). We assume this finding mainly to be explained by our selection process to surgery. No statistically significant difference in mortality between treatment groups at 30, 90, or 365 days was shown, adjusted for age, sex, and ASA class. Comparisons between groups are difficult as our treatment selection, where frailer patients are more likely to undergo nonoperative treatment, introduces selection bias. Also, more factors might affect mortality, making comparisons difficult even after adjustment. However, our results are consistent with earlier studies comparing mortality between operative and nonoperative treatment of acetabular fractures in elderly populations. Walley et al. found no significant difference in 1-year mortality in their study including 87 comorbid elderly patients [23] and a smaller retrospective study showed similar mortality rates between matched cohorts in a healthy elderly population [24]. Our results indicate that mortaility is not directly associated with the choice of treatment. Instead we suggest that we should acknowledge older people with acetabular fractures as frail and strive to mitigate the substantial risks these injuries confer, irrespective of treatment modality. Elderly patients with acetabular fractures have a 30-day mortality comparable to that of hip fractures [25]. Fast-track care and multi-modal care is established in many hospitals for hip fractures and has been shown to reduce mortality and complications [26]. The same attitude of priority and urgency around acetabular fractures could likely improve outcomes.

Posttraumatic osteoarthritis, although without routine long-term radiologic follow-up, was diagnosed in 12 (5%) cases. All cases were found in the operatively treated group and all but 1 of these patients underwent subsequent THA. Our result may be an underestimation due to short-term follow-up. The incidence of osteoarthritis following operative treatment of acetabular fractures has been reported as up to 46% [27] and helped explain a trend towards THA or CHP in the acute setting for certain types of fractures, with characteristics that indicate a poor chance of joint survival. The potential advantage with this strategy is also to enable immediate full weightbearing in accordance with hip fracture mobilization protocols [8,28].

The conversion rate to THA after primary ORIF was 15%, somewhat lower than the 23% conversion rate reported in a systematic review [7]. Secondary treatment for nonoperatively treated patients was surprisingly low (1% during follow-up). We see this as an important finding. It suggests development of osteoarthritis requiring THA conversion in patients initially treated nonoperatively for acetabular fractures is rare. The most important reason for this is probably that this group contains the frailer patients. Future research is needed to further evaluate the development of post-traumatic osteoarthritis and THA conversion rate after nonoperative treatment for acetabular fractures in older populations.

### Strengths

The relatively large sample size (n = 247) is a strength, as well as the linkage to SAR. A comprehensive evaluation of all fractures was conducted, incorporating displacement and impaction variables to determine the severity of each fracture.

### Limitations

The inherent selection bias is a limitation, and the absence of a definitive treatment consensus may lead to treatment choices not being reproducible. Frailer patients and those with undisplaced fractures were more likely to undergo nonoperative management, regardless of fracture type.

There was a gradual shift at our institution during the study period towards the CHP, driven by the recognition of patterns indicative of a bleak prognosis for joint survival solely with ORIF [6]. This shift makes comparisons between different operative treatments difficult.

Mortality data were adjusted for age, sex, and ASA class, but residual confounding likely influenced our results. The result must also be interpreted with caution due to the risk of a type 2 error related to the study design and sample size.

We had some missing data, primarily related to information regarding patients’ residence type and ambulation status before the injury. We may have an underrepresentation of complications, e.g., SSIs and posttraumatic osteoarthritis, as we did not review all records. We could not identify any complications occurring after the patients discharge to the referral hospital. An underrepresentation of complications not leading to secondary surgery is therefore likely. As for posttraumatic osteoarthritis, we were unable to gather this information unless patients underwent a THA conversion due to our lack of a structured protocol for long-term radiographic follow-up.

There is probably a disproportionate distribution towards more complex fracture types and surgically treated patients in this study as patients are referred from other hospitals only in the case of surgery.

A comparison of patient-related outcome measures (PROMs) between operative and nonoperative treatment would have been desirable.

### Conclusion

The 1-year mortality following acetabular fractures in older people was 21% (CI 15–26), underscoring the frailty of this patient group. ORIF alone was associated with a 20% reoperation rate while the rate of delayed surgical treatment in patients selected for nonoperative treatment was 1%.

## Notes

JL: review of medical records and writing. BH, NH, OW: design and supervision. AP: review of medical records. AB: statistical analysis. All authors critically revised the manuscript.

Handling co-editors: Bart A Swierstra

*Acta* thanks Christina Frandsen and Ville Mattila for help with peer review of this manuscript.
